# Development of a New Patient-Reported Outcome to Measure Fatigue in Patients with Multiple Sclerosis

**DOI:** 10.3390/nursrep16030093

**Published:** 2026-03-09

**Authors:** Miguel Angel Jorquera-Ruzzi, Cristina Ramo Tello, Maria José Durà-Mata, Irma Casas

**Affiliations:** 1 Doctoral Program in Biomedical Research and Public Health Methodology, Autonomous University of Barcelona, 08193 Barcelona, Spain; 2 Department of Neurology, 12 de October University Hospital, 28041 Madrid, Spain; 3 Department of Medicine, Autonomous University of Barcelona, 08193 Barcelona, Spain; mjdura.germanstrias@gencat.cat; 4 Department of Physical Medicine and Rehabilitation, Germans Trias I Pujol University Hospital, 08916 Barcelona, Spain; 5 Department of Pediatrics, Obstetrics and Gynecology and Preventive Medicine and Public Health, Autonomous University of Barcelona, 08193 Barcelona, Spain; icasas.germanstrias@gencat.cat; 6 Department of Preventive Medicine and Public Health, Germans Trias I Pujol University Hospital, 08916 Barcelona, Spain

**Keywords:** multiple sclerosis, fatigue, patient-reported outcome measure, nursing, factor analysis, patient-centered care

## Abstract

**Background:** Fatigue is a multidimensional and subjective experience, and it is one of the most common symptoms of multiple sclerosis (MS), affecting up to 80% of patients and acting as a major driver of work disability. Despite its clinical significance, existing assessment tools often lack conceptual clarity or remain too lengthy for routine clinical use. **Objective:** To develop and evaluate a new patient-reported outcome instrument designed to assess multidimensional fatigue domains in patients with multiple sclerosis (MS) for use in clinical practice. **Methods:** This study was carried out in three research stages. Stage 1 (Concept Elicitation) involved qualitative interviews (*n* = 19) to identify core fatigue domains based on patient experience. Stage 2 (Cognitive Interviews) consisted of interviews with 50 patients to ensure the relevance and clarity of the items. Stage 3 (Exploratory Factor Analysis) and internal consistency testing (Cronbach’s alpha) were performed on the same sample of 50 patients to examine the preliminary factor structure and reliability. **Results:** Concept elicitation identified lack of energy and persistent exhaustion as core symptoms. The resulting 14-item instrument covers three subdomains: Psychosocial, Physical, and Cognitive. Exploratory factor analysis supported a three-factor solution explaining 75% of the total variance (Factor 1: 28%; Factor 2: 27%; Factor 3: 20%). Internal consistency was high across all factors: Psychosocial (α = 0.923), Physical (α = 0.895), and Cognitive (α = 0.844). **Conclusions:** This new instrument is a conceptually robust tool that captures the interconnected nature of fatigue in multiple sclerosis (MS). These initial findings support its internal structure and conceptual foundation, providing a practical tool for symptom monitoring in neurological consultations.

## 1. Introduction

Multiple sclerosis (MS) is an autoimmune, inflammatory, and degenerative disease that attacks the myelin of the central nervous system, affecting the transmission of impulses and causing chronic symptoms [1,2]. It is considered one of the most common neurological disorders in young and middle-aged adults [3].

Current estimates indicate that this disease affects more than 2.8 million people worldwide, with a predominant geographical distribution in the northern hemisphere and an incidence in women three times higher than in men [4].

Although the etiology of this disease remains unknown, there is growing evidence pointing to an association between genetic and environmental factors [5,6].

MS can present with a wide range of symptoms, notably motor impairment (muscle weakness, spasticity, ataxia, sensory problems, etc.), cognitive deficits (attention, memory, and executive functions), and psychosocial problems (mood disorders such as depression and/or social difficulties) [7]. In addition, one of the most common and disabling symptoms is fatigue. In fact, more than 80% of people with MS experience fatigue, and its main consequence is limited performance in daily activities [8,9].

The definition of fatigue is sometimes inconsistent and unclear [10], as evidenced by the different concepts used in the literature to define it [11]. One of the most widely accepted reasons for this may be its complex and multidimensional nature, which makes it difficult to conceptualize and measure [12,13].

In an attempt to establish a uniform criterion for defining fatigue, it has been described as an overwhelming feeling of physical and/or mental lack of energy with a disproportionate feeling of tiredness [14,15], which interferes with the performance of daily or desired activities, work, and family/social roles [16,17].

In turn, people with MS describe the symptoms of fatigue as a difficult experience to explain, marked by a sudden onset and constant duration, which does not diminish with rest, characteristics that differentiate it from other types of fatigue [18].

Fatigue in people with MS significantly reduces their ability to perform activities of daily living [8], such as self-care, dressing, going to the bathroom, eating, etc., as well as more complex activities such as communicating, using transportation, shopping, doing household chores, going to work, and socializing, among many other activities [19,20]. This leads to a decrease in these individuals’ well-being and satisfaction, as well as a significant decrease in their quality of life [21,22].

Given its profound impact on quality of life, effective fatigue management is a fundamental goal for MS patients; however, its assessment and measurement present significant challenges [23,24].

In fact, in people with MS, it is difficult to distinguish between fatigue caused directly by the disease and secondary fatigue resulting from comorbidities such as insomnia, depression, or the adverse effects of some medications used to treat MS symptoms [25,26].

The difficulty in managing MS-related fatigue underscores the importance of using comprehensive, validated, and reliable patient-centered assessments to ensure effective treatment [27,28].

Since fatigue in MS is a subjective experience, its assessment benefits from the use of patient-reported outcome (PRO) measures [29].

“PROs, defined by the FDA as any health information about a patient provided directly by the patient, without interpretation by a physician or other health professional, were created to address the above issue” [30].

Currently, there are various PRO instruments used to assess fatigue in MS, both in clinical trials and in clinical practice. However, there is no clear consensus on which is the most appropriate [31,32]. The main reasons for this situation are based on the notable conceptual heterogeneity of the scales that measure fatigue in people with MS [33]. While some focus on the multidimensionality of fatigue, others take a specific domain-specific (unidimensional) approach, which makes it difficult to decide which aspects of fatigue should be included in the process of designing and validating a scale [34]. Furthermore, most of these instruments do not meet the current standards established for the development of PRO instruments [35,36].

Methodological deficiencies in the process of designing and validating PROs are compounded by other obstacles, such as item generation, since the limited implementation of item analysis in the composition of instruments calls into question the reliability of the assessment. Only a few instruments address this situation [37]. Finally, in terms of operationalization, most scales are not suitable for patients with lower educational levels or additional cognitive impairment due to the length and complexity of their sentences [38].

In this context, there is a need for a reliable tool that allows the diagnosis, quantification, and assessment of the main aspects of fatigue from the perspective of people with multiple sclerosis [39,40]. This tool will also help nursing staff to focus their interventions on the aspects of fatigue that most affect patients’ daily well-being. It should facilitate structured dialogue, improve the therapeutic relationship, and enable shared decision-making.

The aim of this study was to identify and describe the characteristics of fatigue from the perspective of people with multiple sclerosis, in order to develop and evaluate a new patient-reported outcome instrument designed to assess multidimensional fatigue domains in patients with multiple sclerosis (MS) for use in clinical practice. The new questionnaire was developed based on qualitative research in the first and second phases, as well as subsequent exploratory factor analysis in the third phase.

## 2. Materials and Methods

**Study Design**

This study employed a sequential mixed-methods instrumental design focused on the development and initial psychometric exploration of a new PROM. The process was conducted in three sequential stages:

**Concept Elicitation (Stage 1)**
**Sampling and Data Collection**

A purposive sampling strategy was employed to ensure a wide representation of fatigue experiences across different levels of disability and disease duration. Individual face-to-face interviews were conducted with 19 participants to identify and describe fatigue-related symptoms and their impact on daily life. Each session lasted approximately 40 min and was guided by a semi-structured interview guide developed by the research team and reviewed by clinical experts. The interviews focused on open-ended questions to explore personal experiences, triggers, and coping mechanisms. All sessions were audio-recorded, transcribed verbatim, and anonymized using ATLAS.ti software, version 25.0.1 (ATLAS.ti Scientific Software Development GmbH, Berlin, Germany).

**Qualitative Analysis and Coding Framework**

The study followed a Thematic Analysis approach. To ensure objectivity, the coding procedure was performed by two independent researchers with expertise in qualitative methodology and multiple sclerosis. This investigator triangulation addressed potential biases (reflexivity), ensuring that the interpretation was grounded in the participants’ data rather than the researchers’ preconceptions. After the initial open coding, an intercoder agreement process was established through consensus meetings to resolve discrepancies and refine the coding framework. This resulted in 9 definitive codes: Activities of daily living, Description of fatigue, Fatigue Measurement, Feeling, Intense moments of fatigue, Sleep quality, Social Interaction, Strategies for coping with fatigue symptoms, and Symptom of fatigue that affects you emotionally.

**Trustworthiness and Saturation**

Following the Standards for Reporting Qualitative Research (SRQR) guidelines [41], several procedures were implemented to ensure trustworthiness. Credibility was established through the direct mapping of patient quotations to concept development. Dependability and confirmability were maintained through a rigorous audit trail, documenting every analytical decision via memos and network diagrams in ATLAS.ti. Conceptual saturation was achieved primarily through the concept elicitation process. Based on existing literature [42], a sample of 17 participants was initially targeted; however, two additional patients were recruited to confirm that no new relevant concepts emerged, thus validating the final sample of 19 participants and ensuring the comprehensiveness of the three subscales: physical, cognitive, and psychosocial.

**Measurement Instrument Format:**

The concepts communicated by patients were selected for inclusion in the initial questionnaire based on how frequently they were mentioned during the interviews. Items were generated based on these concepts (first draft of 14 items), and then the questions were written. The recall period (14 days) was defined, and the order and structure of the questionnaire were established. The range of responses was defined using a Likert scale: “Always, often, sometimes, occasionally, never.”

**Stage 2 Cognitive Interview/Pilot Test**
**Methodology and Iterative Process**

Cognitive interviews and a pilot test were conducted with a new sample of 50 patients with multiple sclerosis who had not participated in the concept elicitation stage. To ensure the content validity of the PROM, we employed a hybrid cognitive interviewing technique, combining “think-aloud” protocols with verbal probing. Participants were asked to verbalize their thought processes while answering the items and were subsequently questioned using specific probes to assess the relevance, comprehensibility, acceptability, completeness, and interpretability of the items, instructions, response options, and the recall period.

**Data Analysis and Modifications**

The interviews were conducted in two iterative waves (25 patients each). This allowed the research team to identify emergent issues in the first wave, implement modifications, and test the refined version in the second wave to ensure that no further comprehension barriers remained. Responses were analyzed using a thematic matrix, where each item was categorized based on the types of difficulties reported by patients (e.g., lexical ambiguity, recall burden, or cultural inappropriateness).

**Refinement Criteria**

An item was flagged for review if at least 20% (*n* = 10) of the respondents provided consistent recommendations to modify or eliminate it due to difficulty in interpretation or comprehensibility. This threshold was not used in isolation; all emergent qualitative feedback from participants was analyzed by the research team to ensure the conceptual integrity of the tool. Items were modified or discarded based on these systematic findings to ensure high face validity and content adequacy prior to conducting an exploratory factor analysis (EFA) to examine the internal structure of the scale.

**Stage 3 Factor analysis**
**Initial Exploration of Dimensionality**

An Exploratory Factor Analysis (EFA) was performed to identify and analyze the dimensions of fatigue, determine the underlying structure of the variables, and evaluate potential item reduction. The suitability of the data for factorization was assessed using Bartlett’s Test of Sphericity (*p* < 0.05) and the Kaiser–Meyer–Olkin (KMO) measure of sampling adequacy (threshold > 0.60) [11,43].

**Factor Extraction and Rotation**

EFA was based on Principal Axis Factoring (PAF), followed by an oblique Oblimin rotation, as the dimensions of fatigue (physical, cognitive, and psychosocial) were theoretically expected to be correlated. The number of factors retained was determined by Kaiser’s criterion (eigenvalues > 1), inspection of the scree plot, and Horn’s parallel analysis [44,45].

**Item Analysis and Reliability**

Factor loadings were interpreted using a threshold of >0.40 [46], and cross-loadings were scrutinized to ensure a simple structure. Internal consistency was assessed using Cronbach’s alpha for both the total scale and its subscales, including 95% confidence intervals. Additionally, item-total correlations were examined to evaluate each item’s contribution. Items showing negative factor loadings were conceptually reviewed and, when justified by their theoretical relevance, were reverse-coded prior to final scoring to ensure directional consistency. Finally, the score distribution was analyzed to assess the scale’s capacity to discriminate across the spectrum of fatigue severity.

**Patient Population**
**Sample Selection and Eligibility Criteria**

Participants were recruited from the outpatient neurology unit at Germans Trias i Pujol Hospital (Barcelona, Spain). The recruitment process consisted of two distinct stages: a Concept Elicitation phase (*n* = 19) and a Cognitive Interviewing phase (*n* = 50)

**Inclusion Criteria**

Patients were eligible if they had a confirmed diagnosis of Multiple Sclerosis (MS) according to the revised McDonald Criteria (RRMS, PPMS, and SPMS subtypes) [47]. Inclusion required participants to be ≥18 years of age, fluent in Spanish, and possess an Expanded Disability Status Scale (EDSS) score of 0 to 6.0 (documented within the past 6 months). Fatigue, the primary construct under study, was defined as a persistent clinical symptom reported by the patient during routine neurological consultations as a factor significantly impacting their quality of life.

**Exclusion Criteria and Confounding Control**

To ensure the specificity of the PRO items to MS-related fatigue, patients were excluded if they presented any comorbid condition capable of independently inducing fatigue (e.g., untreated thyroid dysfunction, severe anemia, or major clinical depression). Additionally, current treatment for autoimmune disorders other than MS or any clinical situation that might interfere with study participation was grounds for exclusion.

**Recruitment Flow and Participation Rates**

A total of 80 eligible patients were invited to participate across both study stages. Of these, 69 patients (86.3%) completed the study. The remaining 11 patients (13.7%) declined to participate, citing a lack of time to attend the required 45 min face-to-face interview.

Data Integrity and Missing Data

The study achieved 100% data completeness among the 69 participants. Due to the supervised, face-to-face nature of the interviews in both the elicitation and cognitive testing phases, researchers ensured that all items were addressed and understood in real-time. Consequently, there were no missing data, and no participants withdrew once the 45 min sessions had commenced.

**Ethical Considerations**

The study was conducted in accordance with the ethical principles for medical research involving human subjects outlined in the Declaration of Helsinki. The study protocol and the development stages of the new PRO were reviewed and approved on 4 November 2024, by the Research Ethics Committee (CEIC) of the Germans Trias i Pujol Hospital, Barcelona, Spain (Ref: CEI PI-24-204). All participants received written information about the study and provided written informed consent prior to participation. The study officially commenced on 17 February 2025.

**Analysis of Population Characteristics**

**Figure 1:**
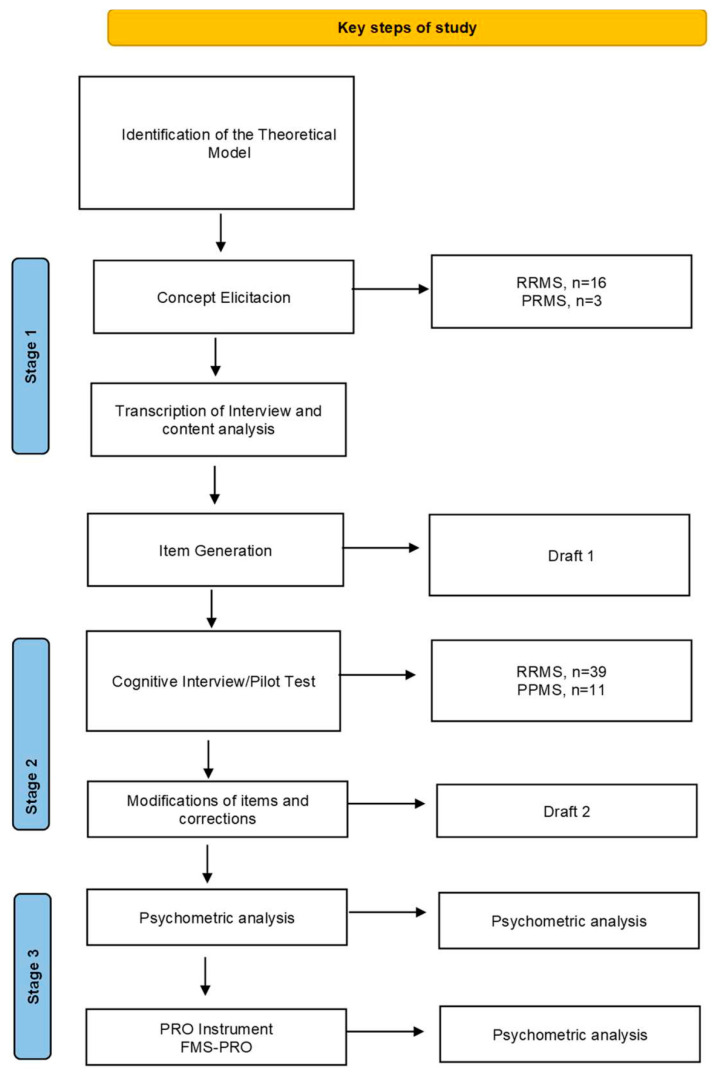
Flow Chart. MS: Multiple Sclerosis; RRMS: Relapsing-Remitting Multiple Sclerosis; PPMS: Primary Progressive Multiple Sclerosis; PRO: Patient-Reported Outcome; EFA: Exploratory Factor Analysis; FMS-PRO: Fatigue Multiple Sclerosis-Patient Reported Outcome.

The characteristics extracted for the descriptive analysis of the population were mean age (standard deviation/range), percentage of females, EDSS score (disease severity), MS subtype (RRMS, SPMS, PPMS), work status, and year of MS diagnosis (Figure 1).

Descriptive analyses of the variables in relation to the participants, as well as psychometric analyses, were carried out using IBM SPSS Statistics (version 27).

## 3. Results

Across the study stages, the participants were predominantly female 38 (55.1%) with an average age of 48.10 ± 11.09 years, ranging from 28 to 68 years of age. The most common subtype was RRMS 55 (78%). The mean EDSS score was 2.48 ± 1.35. Most participants were active full-time workers 50 (72.4%), and the mean year of MS diagnosis was 2013.78 ± 6.99.

The characteristics of the participants are presented in Table 1.

Stage 1: Concept Elicitation Interviews

Concept elicitation interviews were conducted with a total of 19 participants at Germans Trias I Pujol Hospital between January and March 2025, and the analyses showed that saturation was achieved.

The most commonly reported concepts related to fatigue were lack of energy and persistent exhaustion. Participants reported functional impacts due to fatigue in activities of daily living, including difficulties with dressing, using the bathroom, eating, maintaining personal hygiene, and doing household chores.

Other frequently reported concepts included difficulty walking and participating in social and interactive activities, as well as emotional aspects related to fatigue.

The draft of the new patient-reported outcome (PRO) instrument was developed based on the results obtained in the concept elicitation interviews and in accordance with the aforementioned criteria for item generation.

The concepts related to fatigue identified in the concept elicitation stage and the rationale for inclusion and exclusion used in the generation of items in the draft of the new PRO instrument are reported online in Supplementary Material S1.

The development of the instrument also incorporated both evidence from the literature review and contributions from clinical experts specializing in MS treatment.

The new PRO instrument, called FMS-PRO, consisted of 14 items distributed across three conceptual subdomains: physical, cognitive, and psychosocial. The new PRO instrument is presented in Table 2.

The severity and frequency with which the items reflected the concept of fatigue were assessed using a 5-point Likert scale, with response options ranging from “never,” “occasionally,” “sometimes,” “often,” and “always.

The recall period was set at fourteen days (two weeks) to allow for the fact that the concepts assessed may manifest sustained effects over time.

Stage 2: Cognitive interview and Pilot Test

Between May and June 2025, cognitive interviews and pilot testing were conducted with an additional 50 patients who met the same eligibility criteria as those in Stage 1. These interviews took place at Germans Trias i Pujol Hospital, Barcelona, Spain.

They highlighted that it was comprehensive, easy to understand and effectively addressed their major daily challenges related to fatigue.

Furthermore, their contributions and comments improved the clarity of certain terms. Participants also offered suggestions for improving the clarity of some words, and these were addressed through modifications to the initial draft of the questionnaire.

For instance, they defined ‘tiredness’ as a lack of energy resulting from intense activity that is not necessarily linked to MS fatigue. This contrasts with ‘exhaustion’, which they described as a more intense and constant feeling with no apparent cause.

Based on these comments, items that included the term ‘tiredness’ or ‘tired’ were changed to ‘exhaustion’. These items included: item 5, ‘I have felt constantly exhausted for no reason’; item 7, ‘I feel exhausted after doing activities that did not cause me fatigue before’; item 10, ‘I feel exhausted in the mornings despite sleeping well’; and item 11, ‘I experience great exhaustion during the day, but I feel that I cannot rest well at night’. The wording of all these items was revised to specify that the exhaustion experienced by participants was attributable to fatigue. No revisions were required to the recall period or response options. After revision, the PRO instrument retained its initial fourteen items.

Stage 3: Item Performance Analysis

Bartlett’s sphericity test was highly significant (*p* < 0.001), as was the Kaiser–Meyer–Olkin (KMO) measure of sample adequacy (0.85). These results indicate that the correlation matrix was appropriate for exploratory factor analysis. See Table 3.

Exploratory factor analysis based on principal axis factoring, followed by oblimin rotation, revealed three potential subscales from the set of 14 items. All items had factor loadings higher than 0.4 on three factors.

The resulting three-factor solution explained 75% of the total variance. Factor 1 explained 28% of the variance, Factor 2 explained 27%, and Factor 3 explained 20%. Eigenvalues were 7.05, 2.12 and 1.34, respectively. This suggests that the items can be categorized into three conceptual subdomains representing physical, cognitive, and psychosocial aspects. Factor loadings and characteristics are presented in Table 4 and Table 5, respectively. Participants used the full scale, with scores ranging from 0 to 4, and similar distributions were observed for all items.

The internal consistency of the 14-item instrument was evaluated using Cronbach’s alpha coefficients for each of the three identified factors. As shown in Table 6, the scale demonstrated high reliability across all subdomains. The Psychosocial factor showed the highest internal consistency (α = 0.923), followed by the Physical (α = 0.895) and Cognitive (α = 0.844) factors. All coefficients, including their 95% confidence intervals, exceeded the recommended threshold of 0.70, indicating robust internal consistency for this developmental stage.

Analysis of the response distribution revealed that most items obtained high scores, with no ceiling or floor effects. The remaining results of the exploratory factor analysis are presented in online Supplementary Material S2.

## 4. Discussion

The aim of this study was to identify and describe the characteristics of fatigue from the perspective of people with multiple sclerosis in order to develop and evaluate a new patient-reported outcome instrument designed to assess multidimensional fatigue domains in patients with multiple sclerosis (MS) for use in clinical practice.

Fatigue is a prevalent and disabling symptom of MS that negatively impacts quality of life. Due to the inherently subjective nature of this symptom, the most accurate assessment is achieved through patient-reported outcomes. This highlights the importance of having a valid and reliable measurement tool to facilitate the design of optimal, individualized therapeutic strategies.

Despite several PRO instruments being used to assess fatigue in MS, most do not meet current quality criteria. This is partly due to the conceptual imprecision of the construct being measured. Only the NFI-MS and FSQ-RMS patient-reported outcomes instruments meet this criterion.

Based on this premise, this study was conducted using a qualitative approach in its early stages, while rigorously adhering to the current standards for designing a new patient-reported outcome (PRO) measurement instrument [30]. A definition of fatigue was developed based on the literature and the experiences of people with MS who suffer from it [48] through the stages of concept elicitation, cognitive interviews and pilot testing.

The concept elicitation stage enabled us to go beyond the clinical description, capturing the subjective experience of patients and improving our understanding of the real impact of the symptom on their daily lives. This allowed us to identify the specific factors that most concern patients, providing a clear definition of fatigue and enabling us to develop a set of items that effectively reflect this definition.

Participants emphasized the significant impact of fatigue on daily life, noting its effect on both physical and mental capacity. They also expressed frustration at the lack of understanding surrounding their condition, which has an impact on their social interactions and emotional well-being. Despite these challenges, participants demonstrated resilience by maintaining an exercise routine and seeking support. They recognized the importance of adapting to their limitations and striking a balance between their responsibilities and self-care. They emphasized the need for a supportive and understanding environment that allows for rest and recovery.

Conducting the pilot test was essential to ensure the logistical feasibility of the study. The results of this stage enabled ambiguous questions and/or words in the questionnaire to be identified and corrected, significantly improving its interpretability and the response rate among participants, as was the case with changing the word “tiredness” to “exhaustion.”

Participants found the measurement tool easy to use because the instructions were clear and the items were understandable, making the tool dynamic and simple to administer. The paper format was also positively assessed, particularly by older participants. Finally, patients confirmed that the questions in this new PRO measurement tool are relevant to their fatigue and measure meaningful outcomes.

Exploratory factor analysis (EFA) of the scale revealed a factor structure composed of three distinct factors, which demonstrate a solid theoretical correspondence with the critical dimensions of multiple sclerosis. Factor 1 (Psychosocial; items 2, 4, 12, 13, 14) groups elements evaluating the impact of fatigue on social participation and emotional well-being; these items correlate with role restriction and limitations in environmental interaction, capturing how the symptom transcends biological aspects to affect the patient’s relational life. Factor 2 (Physical; items 1, 3, 8, 10, 11) focuses on the somatic manifestations of fatigue, with items measuring the perception of exhaustion and the decline in physical endurance during activities of daily living. Finally, Factor 3 (Cognitive; items 5, 6, 7, 9) describes the mental burden and difficulties in information processing; its items are linked to the self-perception of deficits in attention and concentration, reflecting the mental exhaustion characteristic of the pathology. This alignment between the factor structure and the conceptual domains ensures that the instrument is capable of discriminating the origin and specific impact of fatigue across each area of the individual’s life.

This differentiation suggests that the concept of fatigue at the impact level can be classified into three subdomains. All items in the new measurement instrument had high factor loadings on a single factor. In particular, item 13 had a factor loading of 0.905 on Factor 1, item 11 had a factor loading of 0.930 on Factor 2, and item 5 had a factor loading of 0.865 on Factor 3. These results indicate a strong correlation between the items and their respective latent factors, suggesting that each item clearly measures its respective subdomain. Within the physical subdomain (Factor 2), items 1 and 10 exhibited significant negative loadings (−0.715 and −0.902, respectively). Since the factor structure was optimized using Oblimin rotation, which accounts for the expected correlations between fatigue dimensions, these negative loadings reflect the mathematical orientation of the items within the factor space rather than a conceptual contradiction. Clinically, the high magnitude of these loadings confirms that ‘overwhelming heaviness’ and ‘morning exhaustion’ are core indicators of physical fatigue in this population.

Identifying these three factors has significant theoretical implications. It suggests that the construct of fatigue is more complex than previously thought and reaffirms the concept that fatigue has a multidimensional structure.

This multidimensionality is empirically supported by the factor correlation matrix derived from the Oblimin rotation. The analysis revealed a moderate positive correlation between the Psychosocial and Physical factors (*r* = 0.449), reflecting how somatic limitations directly influence social participation and role restriction. Furthermore, the correlations between the Cognitive factor and the Psychosocial (*r* = −0.391) and Physical (*r* = −0.202) factors, where the negative signs represent the mathematical orientation within the factor space, demonstrate that while these dimensions converge into the global construct of fatigue, they maintain sufficient independence to be evaluated as distinct subdomains.

The relatively low correlation between the physical and cognitive dimensions is particularly noteworthy, as it aligns with clinical observations in Multiple Sclerosis where these manifestations can often dissociate. This statistical evidence ensures that the instrument is capable of discriminating the origin and specific impact of fatigue across each area of the individual’s life, providing a more granular and clinically relevant assessment than unidimensional scales.

The robustness of this tripartite structure is further confirmed by the high levels of internal consistency observed across all subscales. The Psychosocial factor demonstrated excellent reliability (α = 0.923) reflecting a highly cohesive set of items for evaluating social impact. Similarly, the Physical (α = 0.895) and Cognitive (α = 0.844) factors showed strong internal consistency, exceeding the widely accepted threshold of 0.70 for clinical instruments. For the Physical factor, the reliability was calculated by accounting for the reverse-scaled orientation of items 1 and 10, consistent with their negative factor loadings. These findings indicate that the items within each subdomain are not only conceptually aligned but also statistically reliable, ensuring that the instrument provides a stable and precise measurement of the distinct manifestations of fatigue in multiple sclerosis.

Despite these robust psychometric findings, the relatively small sample size (50) requires that results be interpreted with caution, as it may lead to an overestimation of factor stability. While a larger participant-to-item ratio is typically preferred for generalizability, the high magnitude of the observed factor loadings (>0.80 in most cases) and the clear conceptual alignment of the items suggest that the identified tripartite structure is substantial. Consequently, these preliminary findings provide a solid foundation for the instrument, although they must be confirmed with larger sample sizes in future multicenter studies to ensure its performance across the full clinical spectrum of multiple sclerosis.

Although the assumption of unidimensionality measuring a single latent trait through a set of items is a current standard in the design and validation of PROs and a prerequisite for good measures [49], it is difficult to apply in all cases. In the case of fatigue, in particular, achieving this is a complex objective, mainly due to its subjective and multifaceted nature.

Our results are consistent with previous studies [14,50], which suggest that the underlying variable is a multidimensional construct. The emergence of these three specific factors further corroborates the theory that fatigue is multifaceted and multidimensional, represented by a cluster of interrelated aspects that, taken together, are referred to as fatigue and are difficult to measure on a strictly unidimensional level [51].

Currently, under the new standards, only the Design and Validation of the FSIQ-RMS carried out by [52] complies with the unidimensionality assumption in the domain of symptoms. However, a limitation is that the validation of the instrument has not been carried out in people with MS with an EDSS >5.5, so it cannot be said that it is applicable to patients with a more severe condition.

Another instrument that has been used is the PROMIS short form specific to MS [53]. However, correctly applying this bank of items is difficult due to the lack of specification of the subtype of multiple sclerosis to which it is directed, since the symptom of fatigue can vary significantly according to the classification of the disease.

The results of this study should be interpreted with the understanding that fatigue is a multidimensional construct that encompasses physical, mental, and psychosocial aspects which interact with each other in complex ways. Contrary to a simplistic view, fatigue is not solely the result of one aspect, such as a lack of physical energy. Rather, fatigue involves a reduction in the ability to perform tasks effectively due to physical, cognitive, and psychosocial factors. The difficulty concentrating observed in our participants (the cognitive dimension) could have exacerbated the perception of physical effort or exhaustion. This would limit participation in social activities, illustrating the interconnection between these dimensions.

The theoretical need to consider fatigue as a one-dimensional construct, seeking a precise and simple measurement, constitutes an oversimplification that ignores crucial aspects of the experience of fatigue and its varied consequences.

On the other hand, basing the scale on patients’ lived experiences means that the multidimensional model provides a more accurate and nuanced representation of how fatigue is experienced, often leading to an empirical model that demonstrates the multidimensional nature of the construct.

## 5. Strengths and Limitations

One of the main strengths of the study was the rigorous application of current FDA standards [30] in the design stage of a patient-reported outcome measure, incorporating the theoretical conceptualization of fatigue in MS with the experience of patients who suffer from it, ensuring that what really matters to them is measured.

However, our study is not without limitations. One of these is that all participants had an EDSS score < 6; therefore, the results may not reflect relevant aspects of fatigue in individuals with a higher EDSS (>6).

An additional limitation is that, although the inclusion criteria covered all subtypes of MS, the final sample only included patients with the relapsing-remitting and primary-progressive subtypes, as these were the patients who agreed to participate in the study. This means we cannot assume that the concept of fatigue can be extrapolated to other subtypes of the disease.

Furthermore, as this study represents the initial stages of concept elicitation, cognitive interviewing, and exploratory factor analysis within a single-center Spanish cohort, the findings may be subject to cultural bias. While the FMS-PRO reflects the lived experiences of our participants, further cross-cultural validation and linguistic adaptation studies are required to confirm its psychometric stability and generalizability in other Spanish-speaking regions and international contexts.

Finally, it is important to acknowledge that the current study represents an early development stage of the instrument. While our findings provide a preliminary conceptual structure, the sample size (*n* = 50) is relatively small for a definitive exploratory factor analysis, which may lead to an overestimation of factor stability. Consequently, these results should be interpreted as an initial development and exploratory stage rather than a full psychometric validation.

To address these limitations, our research team will proceed to a subsequent research stage focused on a comprehensive validation pathway. This future study will involve a significantly larger sample of patients with multiple sclerosis (MS) to perform confirmatory factor analysis to verify the internal structure and advanced psychometric testing through item response theory to evaluate item precision. Furthermore, this planned research stage will assess the instrument’s temporal stability via test–retest reliability and its longitudinal responsiveness to clinical changes over time. Such a systematic approach is essential to establish the psychometric robustness of the scale across the full spectrum of multiple sclerosis (MS) disability.

In addition, it is suggested that future studies explore the development of fatigue measures for patients with higher disability levels (EDSS > 6.0). Such research is necessary to capture potential dimensions of fatigue unique to more advanced stages of multiple sclerosis (MS) that were outside the scope of the present work.

Implications for clinical practice and research

The use of Patient-Reported Outcome Measures (PROMs) specific to Multiple Sclerosis (MS) fatigue facilitates enhanced monitoring and treatment evaluation, leading to more personalized care. Unlike existing instruments such as the PROMIS Fatigue (MS), which relies on generic item banks, or the NFI-MS, which focuses primarily on functional impact, the FMS-PRO adopts a multidimensional conceptualization (physical, cognitive, and psychosocial) derived directly from patient experiences. The streamlined administration of this tool simplifies data collection, a critical factor for its integration into routine clinical practice. Furthermore, this tripartite structure enables nursing staff and other healthcare professionals to identify specific, often overlooked patient needs. For instance, a high score in the cognitive domain suggests a requirement for neuropsychological rehabilitation, whereas an elevated psychosocial score facilitates early referral to mental health services to address social isolation or stigma. Consequently, these findings allow for comprehensive treatment adjustments that faithfully reflect the unique needs and lived experiences of each patient.

In research, these PROs facilitate the assessment of the effectiveness of new therapies, as they directly quantify fatigue from the patient’s experience and identify subgroups of patients with more pronounced fatigue.

This study presents the first patient-reported outcome measure for fatigue designed specifically for patients with MS in the Spanish language. To date, Spanish-speaking professionals and researchers have relied on translations and validations of instruments developed in other languages, which could lead to cultural and linguistic biases. The aim of this scale is to provide a culturally relevant and linguistically accurate assessment tool for the Spanish-speaking community.

## 6. Conclusions

The results of this study reaffirm that fatigue is an inherently complex and multidimensional construct. Our findings highlight the interconnection of physical, cognitive, and psychosocial factors that manifest themselves in complex ways. The theoretical conceptualization of fatigue remains limited and often confusing. Nevertheless, incorporating the experiences of MS patients who suffer from fatigue into the design of new measurement instruments exponentially helps to improve our understanding of the symptom and the impact it has on people’s lives.

## Figures and Tables

**Table 1:** Patient demographic and clinical characteristics.

Patient Demographic and Clinical Characteristics		
Characteristics	Stage 1:	Stage 2:
	Concept	Cognitive
	elicitation	interviews
	(*n* = 19)	(*n* = 50)
Mean age in years +/− SD	48.58 +/− 10.31	47.90 +/− 11.44
(range)	(28 to 63)	(30 to 68)
Female n (%)	9 (47.4)	29 (58)
Type of MS n (%)		
Relapsing Remitting	16 (84.2)	39 (78)
Primary Progressive		
	3 (15.8)	11 (22)
Secondary		
Progressive		
EDSS, Average, +/− SD	2.47 +/− 1.43	2.48 +/− 1.31
(range),	(1 to 6)	(1 to 6)
EDSS Score n (%)		
1.0	4 (21.1)	5 (10)
1.5	1 (5.3)	6 (12)
2.0	7 (36.8)	20 (40)
2.5	2 (10.5)	2 (4)
3.0	1 (5.3)	5 (10)
4.0	1 (5.3)	2 (4)
4.5	1 (5.3)	2 (4)
5.0	1 (5.3)	4 (8)
5.5	1 (5.3)	2 (4)
6.0	1 (5.3)	2 (4)
Work status n (%)		
Active worker		
(full-time)	15 (78.9)	35 (70)
Active worker		
(part-time)	-	8 (16)
Unemployed	1 (5.3)	1 (2)
Retired	3 (15.8)	6 (12)
Average year of MS diagnosis +/− SD	2013 +/− 7.70	2014 +/− 7.10
(range)	(1997 to 2024)	(1997 to 2024)
Year of MS diagnosis n (%)		
1997	1 (5.3)	1 (2)
1999	1 (5.3)	1 (2)
2002	1 (5.3)	4 (8)
2005	-	2 (4)
2007	1 (5.3)	2 (4)
2008	-	1 (2)
2009	1 (5.3)	1 (2)
2010	1 (5.3)	2 (4)
2012	1 (5.3)	2 (4)
2013	-	2 (4)
2014	1 (5.3)	1 (2)
2015	1 (5.3)	2 (4)
2016	2 (10.5)	4 (8)
2017	1 (5.3)	3 (6)
2018	1 (5.3)	2 (4)
2019	2 (10.5)	5 (10)
2020	2 (10.5)	4 (8)
2021	-	5 (10)
2022	1 (5.3)	4 (8)
2023	-	1 (2)
2024	1 (5.3)	1 (2)

**Table 2:** FMS-PRO, Patient Reported Outcome, Fatigue Multiple Sclerosis-Patient Reported Outcomes, FMS-PRO. Instructions Below is a list of statements describing how fatigue can affect a person. Read each statement carefully and circle the number that best indicates how often fatigue has affected you in this way during the past two weeks. (If you need help marking your answers, tell the interviewer the number of the answer that best fits your situation.) Answer all questions. The interviewer can explain any words or phrases you do not understand.

During the Last Two Weeks					
	Never	Occasionally	Sometimes	Often	Always
1. I feel a sense of overwhelming heaviness.	0	1	2	3	4
2. I find it difficult to describe my fatigue.	0	1	2	3	4
3. I have had difficulty performing my usual daily tasks such as going to work, taking care of the home, or socializing with Friends.	0	1	2	3	4
4. I chose not to go for a walk due to fatigue.	0	1	2	3	4
5. I have felt constantly exhausted for no reason.	0	1	2	3	4
6. I need more rest than usual due to fatigue.	0	1	2	3	4
7. I feel exhausted after doing activities that didn’t used to tire me out.	0	1	2	3	4
8. Physical fatigue prevents me from maintaining a steady pace in my work or daily household activities, and I find it difficult to complete my tasks on time.	0	1	2	3	4
9. I have difficulty concentrating.	0	1	2	3	4
10. I feel exhausted in the mornings despite sleeping well.	0	1	2	3	4
11. I feel very tired during the day, but I feel like I can’t rest well at night.	0	1	2	3	4
12. I have felt more anxious.	0	1	2	3	4
13. I have felt more irritable or angry	0	1	2	3	4
14. I feel that others judge me for not being able to keep up or do the same activities as them because of my fatigue, and this makes me feel uncomfortable.	0	1	2	3	4

**Table 3:** Sample Adequacy Test.

Kaiser–Meyer–Olkin Test		Bartlett’s Test			Chi-Squared Test		
MSA		Χ^2^	df	*p*	Value	df	*p*
Overall MSA	0.848	679.331	91.000	<0.001	Model 117.956	52	<0.001
item10	0.785						
item11	0.817						
item12	0.869						
item13	0.761						
item14	0.822						
item2	0.906						
item3	0.912						
item4	0.941						
item5	0.644						
item6	0.777						
item7	0.862						
item8	0.924						
item9	0.885						
item1	0.785						

**Table 4:** Factor Loadings.

	Factor 1	Factor 2	Factor 3	Uniqueness
item13	0.905			0.311
item4	0.850			0.110
item2	0.751			0.199
item14	0.733			0.150
item12	0.732			0.360
item11		0.930		0.148
item10		−0.902		0.149
item3		0.718		0.159
item8		0.718		0.250
ítem1		−0.715		0.541
item5			0.865	0.281
item6			0.778	0.235
item7			0.721	0.211
item9			0.614	0.386

Note. Applied rotation method is oblimin.

**Table 5:** Factor Characteristics.

Unrotated Solution				Rotated Solution		
	Eigenvalue	Proportion Var.	Cumulative	SumSq. Loadings	Proportion Var.	Cumulative
Factor 1	7.046	0.503	0.503	3.897	0.278	0.278
Factor 2	2.123	0.152	0.152	3.813	0.272	0.551
Factor 3	1.341	0.096	0.096	2.800	0.200	0.751

**Table 6:** Internal consistency.

Factor	Items	Alfa de Cronbach (α)	IC 95%
F1: Psychosocial	2, 4, 12, 13, 14	0.923	[0.882, 0.963]
F2: Physical	1, 3, 8, 10, 11	0.895	[0.851, 0.939]
F3: Cognitive	5, 6, 7, 9	0.844	[0.768, 0.921]

## Data Availability

The data supporting the findings of this study are available from the authors but are subject to restrictions. The authors will consider reasonable requests with prior authorization from the Ethics Committee of Germans Trias i Pujol Hospital, which approved this study’s development.

## Supplementary Materials

The following supporting information can be downloaded at: https://www.mdpi.com/article/10.3390/nursrep16030093/s1, Supplementary Material S1; Concept Elicitation, Codes, Rationale, Supplementary Material S2; EFA, Exploratory Factor Analysis, Supplementary Material S3; Standards for Reporting Qualitative Research (SRQR) guidelines.

## Public Involvement Statement

Patients actively participated in both the concept elicitation and the pilot testing stages.

## Guidelines and Standards Statement

This manuscript was prepared in accordance with the Standards for Reporting Qualitative Research (SRQR) guidelines [41].

## Abbreviations

The following abbreviations are used in this manuscript:

MS	Multiple Sclerosis
RRMS	Relapsing-Remitting Multiple Sclerosis;
PPMS	Primary Progressive Multiple Sclerosis;
PRO	Patient Reported Outcome;
EFA	Exploratory Factor Analysis;
FMS-PRO	Fatigue Multiple Sclerosis- Patient Reported Outcome
