# Supplementary material for: Development of a New Patient-Reported Outcome to Measure Fatigue in Patients with Multiple Sclerosis
Source: Nurs Rep. 2026 Mar 9;16(3):93. doi: 10.3390/nursrep16030093 (PMC13029106; doi:10.3390/nursrep16030093)
Supplement: Supplementary file 1 [file nursrep-16-00093-s001.zip › Supplementary material S1 Concept elicitacion, codes, rationale.pdf]

Supplementary Material S1

Code	Frequency N (%)	Preliminary Decision	Rationale
**Activities of daily living**	550		
● Daily activities include dressing, using the bathroom, eating, maintaining personal hygiene, housekeeping, etc.	372 (67.6)	Include	Concept was frequently reported by participants.
● Difficulties at work	78 (14.18)	Include	Concept was reported with somewhat frequently; most of the patients interviewed are currently working.
● Drive	17 (3.1)	Exclude	Concept was infrequently reported by participants.
● Learning	30 (5.5)	Exclude	Concept was reported infrequently by participants. The term "Learning" has a broad definition and incorporates components that are not directly related to fatigue.
● Leisure and free-time activities	101 (18.2)	Include	Concept was frequently reported by participants.
● Mobility difficulties	99 (18)	Include	Concept was frequently reported by participants.
● Play sports and stay active	26 (4.7)	Exclude	Concept was infrequently reported by participants.
● Public Transportation	20 (3.6)	Exclude	Concept was infrequently reported by participants.
● Sedentary lifestyle	17 (3.1)	Exclude	Concept was infrequently reported by participants. A "sedentary lifestyle" was not only reported as a consequence of fatigue.
● Social activities and/or social interaction	75 (13.6)	Include	Concept was frequently reported by participants.
● Walk	59 (10.7)	Include	Concept was frequently mentioned by participants.

**Table 2. continued**			
● **Description of fatigue**	**837**		
● A feeling that is difficult to explain	2 (0.2)	Exclude	Concept was infrequently reported by participants.
● A symptom that is accentuated in the summer.	45 (5.3)	Include	Concept was frequently reported by participants. This concept was seen by the participants as a key factor in the rise of fatigue.
● A symptom that people who do not suffer from it cannot understand.	8 (0.96)	Exclude	Concept was infrequently reported by participants.
● Anxiety	7 (0.84)	Exclude	Concept was infrequently reported by participants. Anxiety is a term that encompasses multiple components. It is also a medical condition.
● Apathy	6 (0.71)	Exclude	Concept was infrequently reported by participants.
● Confusion	20 (2.39)	Exclude	Concept was infrequently reported by participants.
● Constant concern about tiredness	8 (0.96)	Exclude	Concept was infrequently reported by participants.
● Constant feeling of discomfort	9 (1.1)	Exclude	Concept was infrequently reported by participants.
● Continuous exhaustion	144 (17.2)	Include	Concept was frequently reported by participants.
● Decreased appetite	4 (0.48)	Exclude	Concept was infrequently reported by participants.
● Difficulty in all areas of daily life	115 (13.7)	Include	Concept was frequently reported by participants. This concept is related to that of activities of daily living, which are activities that people perform on a regular basis, such as eating, bathing, and getting dressed.

● Exaggerated effort	45 (5.4)	Exclude	Concept was somewhat frequently reported by participants. However"exaggerated effort" was not only reported as a consequence of fatigue.
● Exhaustion that increases with heat	9 (1.1)	Exclude	Concept was infrequently reported by participants.
● Feeling of doing things out of obligation	11 (1.3)	Exclude	Concept was infrequently reported by participants.
● Feeling of incapacity	47 (5.6)	Include	Concept was somewhat frequently reported by participants.
● Feeling of suffocation	4 (0.5)	Exclude	Concept was infrequently reported by participants.
● Feeling of weakness	17 (2)	Exclude	Concept was infrequently reported by participants.
● Feeling sleepy	2 (0.2)	Exclude	Concept was infrequently reported by participants.
● Fluctuating sensation	11 (1.3)	Exclude	Concept was infrequently reported by participants.
● Intense, internal, and persistent feeling of heaviness	42 (5)	Include	Concept was frequently reported by participants.
● Lack of Energy	42 (5)	Include	Concept was frequently reported by participants.
● Lack of enthusiasm for activities	37 (4.4)	Exclude	Concept was somewhat frequently reported by participants. However, this term, was not only reported as a consequence of fatigue.
● Limiting symptom	88 (10.6)	Include	Concept was frequently reported by participants.
● Loss of attention and concentration	29 (3.46)	Include	Concept was somewhat frequently

			reported by participants.
● Mental and/or psychological fatigue	27 (3.2)	Include	Concept was somewhat frequently reported by participants.
● Mental exhaustion	15 (1.8)	Include	Concept was somewhat frequently reported by participants.
● Not relieved by rest	30 (3.6)	Include	Concept was somewhat frequently reported by participants. This term is directly related to symptoms of fatigue.
● Overwhelm	8 (1)	Exclude	Concept was infrequently reported by participants.
● Overwhelming feeling	47 (5.6)	Include	Concept was somewhat frequently reported by participants. definition of fatigue from the participants' perspective.
● Perception of continuous drowsiness	51 (6.1)	Exclude	Concept was somewhat frequently reported by participants. The perception of continuous drowsiness is not a concept related exclusively to fatigue.
● Physical Exhaustion	85 (10.2)	Include	Concept was frequently reported by participants.
● Recurrent symptom	10 (1.2)	Exclude	Concept was infrequently reported by participants. The term does not provide further information.
● Sensation that prevents you from doing any activity	13 (1.6)	Exclude	Concept was infrequently reported by participants. Concept overlaps with those of activities of daily living. Therefore, they are already

			included for the generation of items.
● Strange sensation	4 (0.5)	Exclude	Concept was infrequently reported by participants. Nonspecific term.
● Stress	10 (1.2)	Exclude	Concept was infrequently reported by participants. The term "stress" includes internal and external factors that are not always directly related to the symptoms of fatigue in people with EM.
● Symptom of difficult Description	211 (25.2)	Include	Concept was frequently reported by participants. This concept highlights the difficulty of defining fatigue.
● Symptom of variable intensity	84 (10)	Include	Concept was frequently reported by participants.
● Symptom that affects you emotionally	21 (2.5)	Exclude	Concept was infrequently reported by participants.
● Symptom that changes your routine	14 (1.7)	Exclude	Concept was infrequently reported by participants.
● Tiredness	287 (34.3)	Include	Concept was frequently reported by participants.
● Uncertainty	12 (1.4)	Exclude	Concept was infrequently reported by participants. Nonspecific term.
● Unexplained feeling of heavy load	66 (7.9)	Include	Concept was somewhat frequently reported by participants.
● Unpleasant Feeling	5 (0.6)	Exclude	Concept was infrequently reported by participants.
**Fatigue Measurement**	**35**		
● Assessment	4 (11.4)	Exclude	Concept was infrequently reported by participants.

● Assessment scales	23 (65.7)	Exclude	Concept was infrequently reported by participants.
● Compilation of experiences	3 (8.6)	Exclude	Concept was infrequently reported by participants.
● Lack of Information	3 (8.6)	Exclude	Concept was infrequently reported by participants.
● Measuring instrument	8 (23)	Exclude	Concept was infrequently reported by participants.
● Questionnaires	23 (65.7)	Exclude	Concept was infrequently reported by participants.
● Self-assessment	1 (2.9)	Exclude	Concept was infrequently reported by participants.
● **Feeling** **1087**			
● Negative	793 (73)	Include	Concept was frequently reported by participants.
● Neutral	193 (18)	Exclude	Concept was infrequently reported by participants.
● Positive	101 (9.3)	Exclude	Concept was infrequently reported by participants.
● **Intense moments of fatigue**	**244**		
● After lunch	5 (2)	Exclude	Concept was infrequently reported by participants.
● After walking	2 (0.8)	Exclude	Concept was infrequently reported by participants.
● Almost always at night	60 (25)	Include	Concept was somewhat frequently reported by participants.
● At dinner time	3 (1.2)	Exclude	Concept was infrequently reported by participants.
● During prolonged activities	4 (1.6)	Exclude	Concept was infrequently reported by participants.
● During the workday	13 (5.3)	Exclude	Concept was infrequently reported by participants.

● During weekends	7 (2.9)	Exclude	Concept was infrequently reported by participants.
● From noon onwards	9 (3.7)	Exclude	Concept was infrequently reported by participants.
● In summer	25 (10.2)	Exclude	Concept was infrequently reported by participants. This concept overlaps with that of increased fatigue in summer, which has been included for the generation of items.
● In the evening	45 (18.4)	Exclude	Concept was infrequently reported by participants.
● Long car journeys	7 (2.9)	Exclude	Concept was infrequently reported by participants.
● The intensity is constant.	97 (39.8)	Include	Concept was frequently reported by participants.
● Throughout the Day	55 (22.5)	Exclude	Concept was somewhat frequently reported by participants.
● When going to bed	4 (1.6)	Exclude	Concept was infrequently reported by participants.
● when I do any of my daily activities	25 (10.2)	Exclude	Concept was infrequently reported by participants.
● When I wake up in the morning, whatever time it is	83 (34)	Include	Concept was frequently reported by participants.
● When I want to go to sleep	14 (5.7)	Exclude	Concept was infrequently reported by participants.
● **Sleep quality**	**129**		
● Despite being tired, I can't sleep well.	36 (27.9)	Include	Concept was frequently reported by participants.
● Difficulty falling asleep	18 (14)	Exclude	Concept was somewhat frequently reported by participants. This term

			encompasses multiple components.
● Difficulty resting	3 (2.3)	Exclude	Concept was infrequently reported by participants.
● Insomnia	27 (21)	Exclude	Concept was infrequently reported by participants. The term insomnia encompasses multiple components.
● Interrupted sleep during the night	66 (51)	Include	Concept was frequently reported by participants. However, this term is unspecific.
● Non-restorative rest	15 (11.6)	Exclude	Concept was infrequently reported by participants.
● Non-Restorative Sleep	13 (10.1)	Exclude	Concept was infrequently reported by participants.
● Poor sleep quality	44 (34)	Exclude	Concept was frequently reported by participants. However, this term is unspecific.
● Tiredness upon waking	14 (10.9)	Exclude	Concept was infrequently reported by participants.
● **Social Interaction**	**203**		
● Communication difficulties	1 (0.5)	Exclude	Concept was infrequently reported by participants.
● Decline in social life	3 (1.5)	Exclude	Concept was infrequently reported by participants.
● Difficulty concentrating	3 (1.5)	Exclude	Concept was infrequently reported by participants. This term was already addressed in the item "Description of fatigue".
● Difficulty initiating conversation	6 (3)	Exclude	Concept was frequently reported by participants. This term has a direct relationship as a consequence of fatigue.

● Difficulty interacting with people	14 (6.9)	Include	Concept was frequently reported by participants. This term has a direct relationship as a consequence of fatigue.
● Difficulty participating in group activities	6 (3)	Exclude	Concept was infrequently reported by participants.
● Difficulty paying attention in conversations	3 (1.5)	Exclude	Concept was infrequently reported by participants.
● Exhaustion	4 (2)	Exclude	Concept was infrequently reported by participants.
● Feeling guilty about not participating in family or friend gatherings	2 (1)	Exclude	Concept was infrequently reported by participants.
● Frustration at not being able to share with others	5 (2.5)	Exclude	Concept was infrequently reported by participants.
● Inability to participate in social activities	4 (2)	Exclude	Concept was infrequently reported by participants.
● Irritability due to not participating in family events	6 (3)	Include	Concept was frequently reported by participants.
● Isolation	26 (13)	Include	Concept was frequently reported by participants. Participants highlighted that this term was a direct consequence of fatigue.
● Lack of communication	3 (1.5)	Exclude	Concept was infrequently reported by participants.
● Lack of motivation to participate in social activities	2 (1)	Exclude	Concept was infrequently reported by participants.
● Lack of social support	2 (1)	Exclude	Concept was infrequently reported by participants.
● Limited participation	14 (6.9)	Include	Concept was frequently reported by participants.
● Loss of communication skills	19 (9.4)	Include	Concept was frequently reported by participants.

● Misunderstandings	15 (7.4)	Exclude	Concept was infrequently reported by participants.
● Need to rest after meeting with family or friends	4 (2)	Exclude	Concept was infrequently reported by participants.
● not in the mood to socialize	8 (3.9)	Exclude	Concept was infrequently reported by participants.
● Preference for staying at home	8 (3.9)	Exclude	Concept was infrequently reported by participants.
● problems in family interaction	6 (3)	Exclude	Concept was infrequently reported by participants.
● Reduced interaction	7 (3.4)	Exclude	Concept was infrequently reported by participants.
● Social apathy	7 (3.4)	Exclude	Concept was infrequently reported by participants.
● Social avoidance	13 (6.4)	Exclude	Concept was infrequently reported by participants.
● Social fatigue	10 (4.9)	Exclude	Concept was infrequently reported by participants.
● Social Limitation	53 (26.1)	Include	Concept was somewhat frequently reported by participants.
● Social Prejudice	9 (4.4)	Exclude	Concept was infrequently reported by participants.
● Stress	3 (1.5)	Exclude	Concept was infrequently reported by participants.
● Uncomfortable interaction with others due to their lack of understanding of my situation	101 (50)	Include	Concept was frequently reported by participants.
● **Strategies for coping with fatigue symptoms**	**176**		
● Acceptance	4 (2.3)	Exclude	Concept was infrequently reported by participants.
● Adaptation	12 (6.8)	Exclude	Concept was somewhat frequently reported by participants. However, this term is unspecified.

● Changes in eating habits	2 (1.1)	Exclude	Concept was infrequently reported by participants.
● Coping	3 (1.7)	Exclude	Concept was infrequently reported by participants.
● Coping strategies	31 (17.7)	Exclude	Concept was frequently reported by participants.<br>However, this term is unspecified.
● Energy recovery through prolonged rest	6 (3.4)	Exclude	Concept was infrequently reported by participants.
● Family support	6 (3.4)	Exclude	Concept was infrequently reported by participants.
● force yourself to do activities	15 (8.5)	Include	Concept was frequently reported by participants.
● Improve sleeping habits	9 (5.1)	Exclude	Concept was infrequently reported by participants.
● Increase healthy habits	5 (2.8)	Exclude	Concept was infrequently reported by participants.
● Increased disease awareness	4 (2.3)	Exclude	Concept was infrequently reported by participants. "Increased disease awareness is a term related to multiple sclerosis in general, but not to fatigue specifically".
● Information Search	3 (1.7)	Exclude	Concept was infrequently reported by participants.
● Knowing myself and knowing what I can and cannot do	35 (20)	Include	Concept was frequently reported by participants.
● Medication	6 (3.4)	Exclude	Concept was infrequently reported by participants.
● Personal Effort	4 (2.3)	Exclude	Concept was infrequently reported by participants.

● Plan things further in advance	21 (12)	Exclude	Concept was infrequently reported by participants.
● Positive attitude towards adversity	4 (2.3)	Exclude	Concept was infrequently reported by participants.
● Prioritize Rest	47 (27)	Include	Concept was frequently reported by participants.
● Professional Support	4 (2.3)	Exclude	Concept was infrequently reported by participants.
● Psychotherapy	2 (1.1)	Exclude	Concept was infrequently reported by participants.
● Reading	6 (3.4)	Exclude	Concept was infrequently reported by participants.
● Reduce physical activity	14 (8)	Exclude	Concept was infrequently reported by participants.
● Relaxation	11 (6.3)	Exclude	Concept was infrequently reported by participants.
● Resuming physical exercise	14 (8)	Exclude	Concept was infrequently reported by participants.
● self-motivate	4 (2.3)	Exclude	Concept was infrequently reported by participants.
● try to distract yourself	2 (1.1)	Exclude	Concept was infrequently reported by participants.
● Walk	7 (4)	Exclude	Concept was infrequently reported by participants.
● Writing	2 (1.1)	Exclude	Concept was infrequently reported by participants.
● **Symptom of fatigue that affects you emotionally**	**138**		
● Anger	6 (4.3)	Exclude	Concept was infrequently reported by participants.
● Anxiety	5 (3.6)	Exclude	Concept was infrequently reported by participants.
● Constant mood swings	80 (58)	Exclude	Concept was frequently reported by participants.

● Crying	1 (0.7)	Exclude	Concept was infrequently reported by participants.
● Decay	6 (4.3)	Exclude	Concept was infrequently reported by participants.
● Demotivation	3 (2.2)	Exclude	Concept was infrequently reported by participants.
● Depression	9 (6.6)	Exclude	Concept was infrequently reported by participants. Depression is a term that encompasses multiple components. It is also a medical condition.
● Distress	1 (0.7)	Exclude	Concept was infrequently reported by participants.
● Emotional fatigue	3 (2.2)	Exclude	Concept was infrequently reported by participants. This term overlaps with other concepts already included.
● Emotional fluctuation	24 (17.4)	Include	Concept was frequently reported by participants.
● Feeling of guilt	2 (1.4)	Exclude	Concept was infrequently reported by participants.
● Frustration	5 (3.6)	Exclude	Concept was infrequently reported by participants.
● Futility	2 (1.4)	Exclude	Concept was infrequently reported by participants.
● Irritability	23 (17)	Include	Concept was frequently reported by participants. Participants highlighted that this term was a direct consequence of fatigue.
● Isolation	7 (5.1)	Exclude	Concept was infrequently reported by participants.

● Mental fatigue	15 (11)	Include	Concept was infrequently reported by participants.
● Mood Fluctuation	86 (62.3)	Include	Concept was frequently reported by participants.
● Nervousness	2 (1.4)	Exclude	Concept was infrequently reported by participants.
● Psychological Fatigue	2 (1.4)	Exclude	Concept was infrequently reported by participants.
● Rage	2 (1.4)	Exclude	Concept was infrequently reported by participants.
● Sadness	3 (2.2)	Exclude	Concept was infrequently reported by participants.
