# Supplementary material for: Development of a New Patient-Reported Outcome to Measure Fatigue in Patients with Multiple Sclerosis
Source: Nurs Rep. 2026 Mar 9;16(3):93. doi: 10.3390/nursrep16030093 (PMC13029106; doi:10.3390/nursrep16030093)

Supplementary material S2. Results of exploratory factor analysis.

Factor Loadings (Structure Matrix)

	Factor 1	Factor 2	Factor 3
Item 10	-0.428	-0.908	
Item 11	0.402	0.923	
Item 12	0.779	0.495	
Item 13	0.816		
Item 14	0.862		-0.637
Item 2	0.874	0.501	-0.484
Item 3	0.646	0.866	
Item 4	0.934	0.484	-0.472
Item 5			0.796
Item 6	-0.477		0.849
Item 7	-0.571		0.838
Item 8	0.574	0.833	
Item 9	-0.431	-0.489	0.698
Item 1		-0.607	

Note. Applied rotation method is oblimin.

Factor Correlations

	Factor 1	Factor 2	Factor 3
Factor 1	1.000	0.449	-0.391
Factor 2	0.449	1.000	-0.202
Factor 3	-0.391	-0.202	1.000

Parallel Analysis.

	Real data factor eigenvalues	Simulated data mean eigenvalues
Factor 1*	6.832	1.186
Factor 2*	1.746	0.785
Factor 3*	1.064	0.593
Factor 4	0.124	0.429
Factor 5	0.038	0.281
Factor 6	-0.084	0.151
Factor 7	-0.149	0.056
Factor 8	-0.225	-0.055
Factor 9	-0.278	-0.133
Factor 10	-0.281	-0.223
Factor 11	-0.409	-0.327
Factor 12	-0.431	-0.406
Factor 13*	-0.472	-0.517
Factor 14	-0.642	-0.636

Note. '*' = Factor should be retained. Results from FA-based parallel analysis.

Scree plot

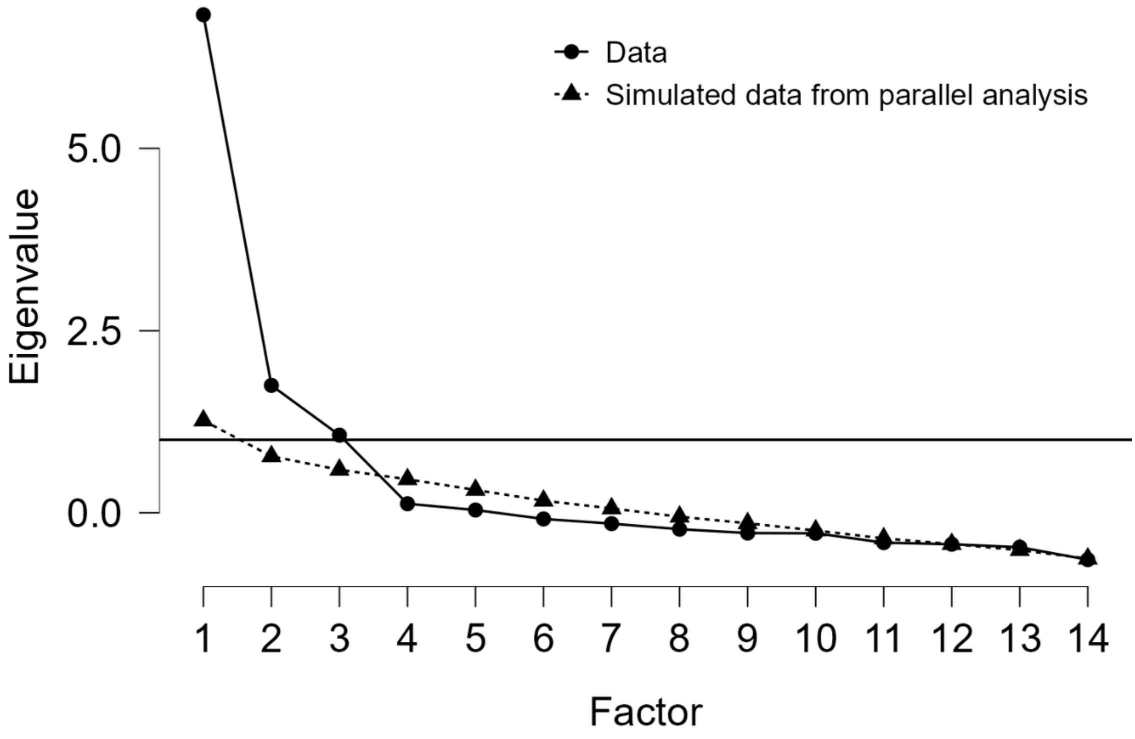

Supplement: Supplementary file 1 [file nursrep-16-00093-s001.zip › Supplementary material S2, EFA.pdf]
